# Disseminated Tuberculosis Presenting as Cerebellar Dysfunction and Adrenal Insufficiency in an Immunocompetent Patient: A Rare Coexistence

**DOI:** 10.7759/cureus.30551

**Published:** 2022-10-21

**Authors:** Wajeeha Batool, Sulhera Khan, Shabnam Naveed, Syed Masroor Ahmad

**Affiliations:** 1 Department of Internal Medicine, Jinnah Postgraduate Medical Centre, Karachi, PAK; 2 Department of Internal Medicine, Jinnah Sindh Medical University, Karachi, PAK

**Keywords:** glucocorticoid replacement, antituberculous treatment, addison’s disease, cerebellar tuberculoma, disseminated tuberculosis

## Abstract

Disseminated tuberculosis is more prevalent in immunocompromised hosts; however, it can affect people with intact immune systems. Here, we present a case of an immunocompetent young woman who presented with headache, vomiting, and dizziness for the past two months. There was a history of significant weight loss during this period. Examination revealed postural hypotension and positive cerebellar signs. Imaging of the brain revealed a conglomerate mass in the cerebellar vermis suggestive of tuberculoma. Tubercle bacilli were detected in gastric lavage specimens. Laboratory investigations revealed hyponatremia with low serum osmolality. Further investigations showed low serum cortisol and high adrenocorticotrophic hormone levels. CT of the abdomen revealed atrophy of both adrenal glands. Our patient was diagnosed with cerebellar dysfunction and adrenal insufficiency secondary to disseminated tuberculosis. We started the patient on antituberculous drugs, along with mineralocorticoid and glucocorticoid replacement. Subsequent follow-up showed significant improvement in symptoms. Hence, timely diagnosis of the disease is essential to prevent lethal outcomes.

## Introduction

Tuberculosis (TB) is one of the most prevalent infectious disorders in the world, affecting 5.6 million men, 3.3 million women, and 1.1 million children worldwide [1]. TB is the 13th leading cause of death and the second biggest infectious cause of death after coronavirus disease 2019 (COVID-19) [1]. Worldwide, among the countries with the highest burden of TB, Pakistan ranks at number five. There are 348,276 cases reported annually, with a mortality of 34 per 100,000 [2]. The most common cause of Addison’s disease is the autoimmune destruction of the adrenal glands; however, infectious causes remain the most common cause of primary Addison’s disease in developing countries. The infections leading to adrenal insufficiency include sepsis, TB, HIV, disseminated fungal infections, cytomegalovirus, syphilis, and histoplasmosis, among which HIV and TB are the most common [3].

Similarly, TB may manifest in the central nervous system, involving the extra- and/or intra-axial compartments [4]. Among these presentations, the most frequent are tuberculous meningitis and tuberculoma, both of which commonly affect immunocompromised hosts [4]. However, in an immunocompetent host, differentiating a tuberculoma from other causes of space-occupying lesions is challenging and requires expertise and effort [5]. Here, we present a case of disseminated TB in an immunocompetent patient with unusual presenting complaints of tuberculous adrenalitis and cerebellar tuberculomas.

## Case presentation

A previously healthy 25-year-old unmarried woman, a resident of Karachi, Pakistan, presented to the general medicine ward via the outpatient department with chief complaints of headache and intractable vomiting for the past two months. Headache was of a dull aching nature and moderate intensity, involving the entire cranium and worse on awakening. The patient, however, did not notice any obvious change in vision, nor did she develop seizures or gross focal deficits. Likewise, no other gastrointestinal symptoms were associated with vomiting. On further questioning, the patient mentioned that she had been experiencing dizziness, which was worst when standing up. The patient had an unexplained weight loss of 11% over two months. The patient denied fever, night sweats, cough, chest pain, and shortness of breath. There was no previous history of similar symptoms, and her past medical history was uneventful as was her family history. She also denied sick contacts, mainly exposure to TB, or the use of immunosuppressive medications.

Upon examination, she was alert and well-oriented but anxious, with a body mass index of 16.2 kg/m2. Blood pressure was 94/60 mm Hg in the lying down position, with a postural drop to 72/56 mmHg on standing up. The patient's heart rate was 110 beats per minute with a regular rhythm, and her respiratory rate was 16 breaths per minute. Random blood sugar was 68 mg/dl. She was afebrile and had mild anemia. No lymphadenopathy was found on examination. The patient had generalized hyperpigmentation, more marked over the axillae, neck, and oral mucosa. Examination of the central nervous system found positive cerebellar signs, mainly truncal ataxia, bilateral horizontal nystagmus, dysmetria (i.e., impaired pass-pointing and impaired heel-shin coordination), and difficult tandem walking. Fundoscopy revealed grade 1 papilledema in both eyes; however, both eyes had normal visual acuity, and peripheral vision was also normal in all four quadrants of both eyes. All other cranial nerves and motor and sensory systems were intact. Higher mental functions were normal. There were no signs of meningism, and both pupils were equal and reactive to light. Ear examination was also unremarkable. Chest auscultation revealed occasional scattered coarse crackles. Succussion splash was not present upon abdominal examination. Cardiovascular and rheumatologic examinations were unremarkable.

**Figure 1:**
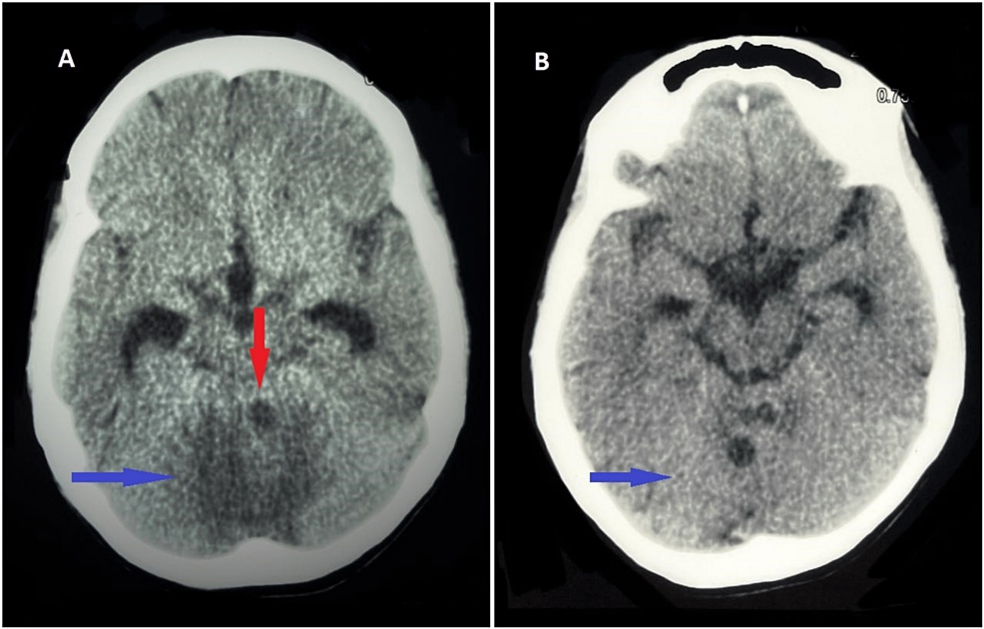
CT scan of the brain (plain) A: Axial view showing hypodense lesion (red arrow) in cerebellar vermis with significant perilesional edema (blue arrow). B: Axial view showing significant resolution of perifocal edema (arrow) two weeks after initiating the treatment.

**Figure 2:**
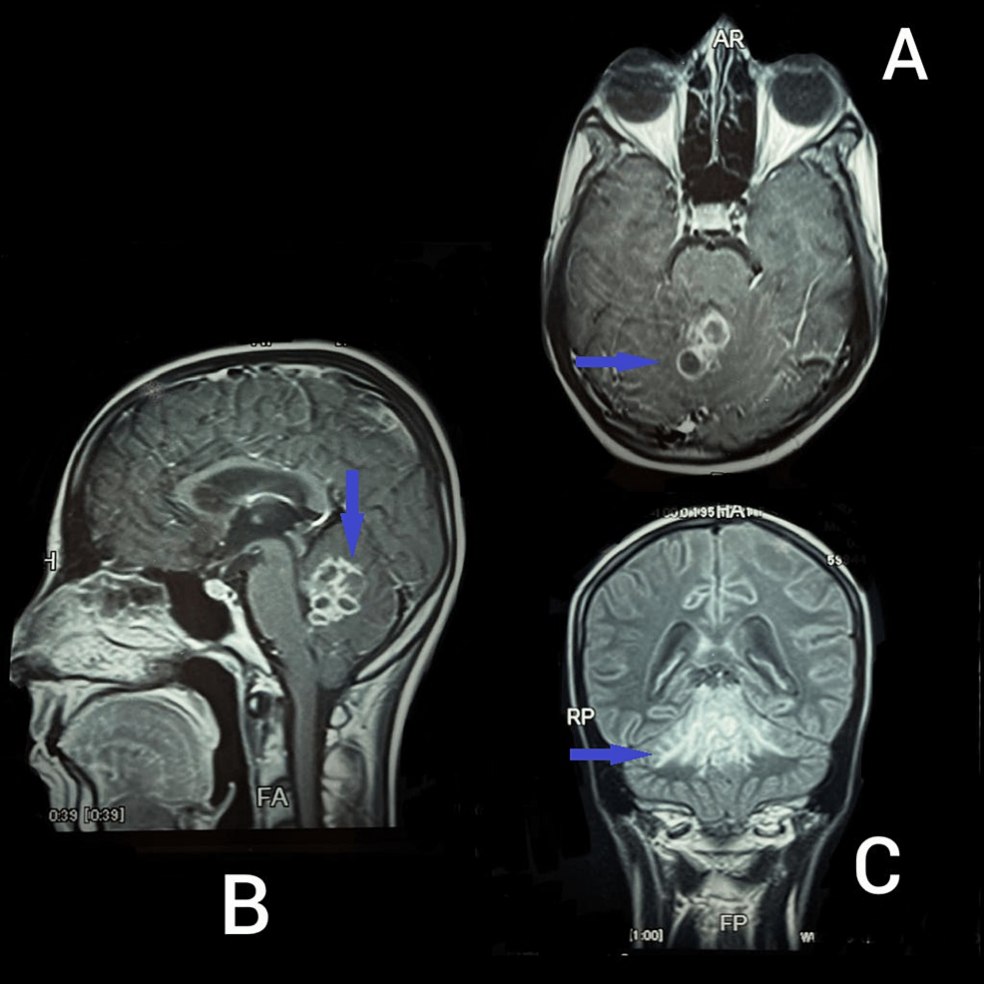
MRI of the brain A: Axial view showing peripheral ring-enhancing confluent lesions (arrow) in cerebellar vermis on post-contrast imaging. B: Sagittal view showing conglomerate mass (arrow) seen in cerebellum on post-contrast imaging. C: Lesions appearing hyperintense on fluid-attenuated inversion recovery (FLAIR) imaging with surrounding edema (arrow) in coronal view.

**Figure 3:**
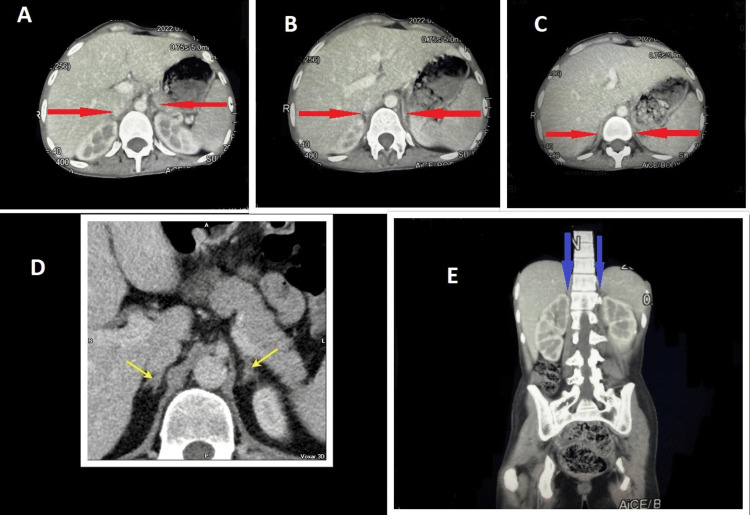
CT scan of the abdomen with contrast A-C: Multiple axial sections showing atrophied adrenal glands, not being visualized clearly (arrows). D: Normal CT anatomy of adrenal glands. The image is taken from Radiopaedia for comparison [6]. E: Glands not visible at their normal anatomic position, indicating atrophy.

Initial laboratory investigations (Table 1) reported anemia (hemoglobin: 10.0 g/dl) with low mean corpuscular volume (76), hyponatremia (126 mEq/l) with normal potassium (4.2 mEq/l), low bicarbonate (16 mmol/l), and an elevated erythrocyte sedimentation rate (ESR; 88 mm/hr). CT of the brain without contrast was done to look for any space-occupying lesions causing these symptoms (Figure 1). It showed small hypodense lesions in the cerebellar vermis with significant surrounding edema (Figure 1A). Subsequent magnetic resonance imaging of the brain (with contrast) revealed a conglomerate mass (anteroposterior × transverse × craniocaudal: 2.4 × 2.0 × 2.7 cm) centered over the fourth ventricle cerebellar vermis causing mild proximal hydrocephalus along with significant perilesional edema (Figure 2). These lesions were suggestive of tuberculomas. Because of the risk associated with a lumbar puncture with a posterior fossa mass, cerebrospinal fluid (CSF) analysis was not performed. CT of the chest showed multiple patches of fluffy infiltrates with segmental thickening and ground glass haze along with bilateral calcified hilar lymph nodes. Tubercle bacilli were detected in two specimens of gastric lavage (this sample was taken because the patient did not produce sputum even after nebulization with hypertonic saline). An HIV serology test was negative. During her hospital stay, the patient had frequent attacks of hypoglycemia despite dextrose infusion. Serum osmolality was also low, i.e., 258 mOsm/Kg H2O. Urinary studies were conducted to look for the cause of hyponatremia, which was not improving with saline hydration. The studies revealed raised urine osmolality (448 mOsm/kg H2O) with high urinary sodium, i.e., 36 meq/L. These findings (along with hypoglycemia, hypotension, hyperpigmentation, and metabolic acidosis) were more suggestive of underlying adrenal insufficiency causing hyponatremia rather than the syndrome of inappropriate antidiuretic hormone secretion (SIADH) secondary to CNS disease. Further investigations were conducted in this regard, with 9 am serum cortisol low at 2.0 mcg/dL and adrenocorticotropic hormone (ACTH) high at 258.2 pcg/mL. This was consistent with the diagnosis of primary adrenal insufficiency most likely due to TB. A CT scan of the abdomen with intravenous contrast revealed bilateral adrenal atrophy without evidence of calcification (Figure 3). An adrenal biopsy was not performed because the presentation was highly suggestive of TB adrenalitis with active extra-adrenal TB.

**Table 1:** Laboratory results for blood tests ACTH = adrenocorticotropic hormone; Alk phos = alkaline phosphatase; ALT = alanine aminotransferase; AST = aspartate aminotransferase; BUN = bun urea nitrogen; ESR = erythrocyte sedimentation rate; GGT = gamma-glutamyl transferase; Hb = hemoglobin; HCT = hematocrit; PLT = platelets; MCV = mean corpuscular volume; WBC = white blood cells.

Complete blood count		On admission	Day 7
Hb		10.0 g/dl	9.6 g/dl
MCV		72.3 fl	74.2 fl
HCT		32.4%	30.2%
PLT		274 * 10*9/ L	315 * 10*9/L
WBC		6.23 * 10*9/L	5.8 * 10*9/L
Neutrophil		82%	84%
Lymphocyte		11.5%	10%
Eosinophil		0.2%	0.2%
Monocyte		6.2%	5.7%
Basophil		0.1%	0.1%
ESR		88 mm/hr	72 mm/hr
Basic metabolic panel			
Sodium	126 mEq/L		122 mEq/L
Potassium	4.2 mEq/L		4.8 mEq/L
Chloride	100 mEq/L		98 mEq/L
Bicarbonate	16 mmol/L		18 mmol/L
BUN	8 mg/dl		9.2 mg/dl
Creatinine	0.50 mg/dl		0.30 mg/dl
Glucose	65 mg/dl		56 mg/dl
Calcium	8.8 mg/dl		8.5 mg/dl
Magnesium	2.2 mg/dl		2.1 mg/dl
Phosphorus	4.0 mg/dl		3.8 mg/dl
Serum osmolality	258 mOsm/kg H2O		250 mOsm/kg H2O
Urine osmolality	448 mOsm/kg H2O		456 mOsm/kg H2O
Hepatic function			
Total bilirubin	0.53 mg/dl		0.48 mg/dl
Direct bilirubin	0.02 mg/dl		0.05 mg/dl
Indirect bilirubin	0.51 mg/dl		0.43 mg/dl
ALT	33 U/L		36 U/L
AST	28 U/L		24 U/L
Alk phos	99 U/L		105 U/L
GGT	16 U/L		19 U/L
Total protein	6.3 mg/dl		6.4 mg/dl
Albumin	2.9 mg/dl		3.0 mg/dl
Globulin	3.4 mg/dl		3.4 mg/dl
9 am serum cortisol level	2.0 mcg/dl (normal: 5-23 mcg/dl)		
ACTH levels	258.2 pcg/ml (normal: 10-60 pcg/ml)		

Based on the abovementioned investigations and clinical findings, the patient was diagnosed with disseminated TB involving the lungs, cerebellum, and adrenal glands. She was started on multidrug treatment for TB, including rifampicin, isoniazid, ethambutol, and pyrazinamide for two months followed by rifampicin and isoniazid for an additional 10 months. The patient was also treated with intravenous dexamethasone (0.3 mg/kg for two weeks followed by 0.2 mg/kg for one week, 0.1 mg/kg for one week, and then tapering oral steroids in a maintenance dose). Fludrocortisone (0.1 mg/day) was also given to build up blood pressure. There was an improvement in her symptoms with steroid replacement. She was then discharged with close follow-up. The patient is improving with treatment. Follow-up imaging (non-contrast brain CT) after two weeks of initiating treatment showed significant resolution of edema surrounding the tuberculoma (Figure 1B).

## Discussion

Disseminated TB is potentially a fatal condition, and a person is said to have disseminated infection if there is histopathologic evidence or detection of tubercle bacilli in bone marrow, liver, bloodstream, or two or more noncontiguous organs, or there is miliary TB. Diagnosis remains a challenge because of the wide variety of presentations depending upon many factors such as immunity of the patient, coexisting diseases, site of involvement, and severity of the disease [4]. In our case, the patient had disseminated TB involving the adrenals, cerebellum, and lungs: the three noncontagious organs. There are several risk factors for the dissemination of TB, such as concomitant HIV infection, malnutrition, alcoholism, organ transplant, or the use of immunosuppressive agents [4]. However, our patient did not have any of these risk factors, suggesting that the dissemination may occur in an immunocompetent individual.

Central nervous system TB can present as tuberculous meningitis, tuberculoma, or rarely as focal cerebritis or abscesses. Clinical presentation of tuberculomas depends upon the site of involvement; in our case, the patient presented with cerebellar dysfunction. Tuberculomas can also produce symptoms of mass effect (headache, vomiting, seizures, and papilledema) that mimic space-occupying intracranial masses. Cerebellar tuberculomas impose a greater risk of obstructive hydrocephalus because of the close anatomic relationship of the cerebellum with the fourth ventricle. They usually appear as hypodense or isodense mass lesions on non-contrast CT studies, but can be observed as solid or ring-enhancing lesions on contrast images [5], while their appearance on MRI depends upon their stage of maturation and whether they are caseating or noncaseating [5].

Our patient also suffered from adrenal involvement of TB seen as Addison’s disease, which resulted in caseous necrosis and subsequent atrophy of adrenal glands. Studies have shown that at least 90% of the adrenal parenchyma must be destructed before patients develop adrenal insufficiency. This may explain why most patients with tuberculous adrenalitis have no improvement in their insufficiency even after antituberculous drug therapy [7]. CT imaging of adrenal glands shows disease at various stages. In the early stages of active infection, there is a mass-like enlargement of glands while atrophied glands and calcification suggest late disease or remote infection [3]. Although our patient had an active infection outside the adrenals, atrophy of the glands leading to adrenal insufficiency was intriguing of the hypothesis that our patient had developed primary TB several years ago (evident by calcified hilar lymph nodes in the CT chest scan), which ultimately resulted in Addison’s disease. Nomura et al. observed that 93% of the patients with adrenal TB had previous extra-adrenal TB, and the mean period from previous non-adrenal TB to adrenal TB was 31.9 years [8]. Hence, imaging features help estimate the clinical duration of Addison’s disease and in providing a guide to clinicians on a management plan. The goal of treatment is to replace adrenal hormones to improve blood pressure, correct electrolyte abnormalities, and reverse cortisol deficiency. However, care must be taken while treating TB and Addison’s disease simultaneously, because rifampicin is a potent inducer of the cytochrome P450 enzyme, which is involved in the metabolism of adrenal hormones. A study by McAllister et al. revealed that rifampicin increases the plasma clearance of prednisolone by up to 45% and reduces the amount of drug available to the tissues (area under the plasma concentration-time curve) by 66%. Therefore, it is advised to increase the dose of steroids when starting them concomitantly with rifampicin, with their subsequent dose reduction on stopping the drug [9] to prevent a potentially life-threatening Addisonian crisis as a result of treatment. We treated our patient with antituberculous drugs, fludrocortisone (to augment blood pressure) and dexamethasone (given in the tapering dose recommended for CNS TB and preferred over other glucocorticoids because of potent anti-inflammatory properties). The plan is to continue glucocorticoid and mineralocorticoid replacement therapy, with the lowest effective dose possible.

## Conclusions

Our case illustrates that TB can present with a wide variety of symptoms depending on the site involved. Symptoms of mass effect can be the first presentation of CNS tuberculomas, mimicking space-occupying intracranial lesions. Thus, tuberculomas are an important differential to consider in patients presenting with signs of raised intracranial pressure. Likewise, Addison's disease secondary to TB should be considered in patients with signs of adrenal insufficiency, especially those with concomitant active tuberculous (pulmonary or extrapulmonary). Imaging helps determine whether adrenal insufficiency is secondary to active disease or a remote process. Hence, early diagnosis and treatment of TB can save patients from a chronic yet fatal disease and its complications.
